# Auspice and other policy-related variations in preschool practice in the United States: have public preschool programs been more academic?

**DOI:** 10.1186/s40723-024-00139-6

**Published:** 2024-12-05

**Authors:** W. Steven Barnett, Kwanghee Jung

**Affiliations:** National Institute for Early Education Research, Rutgers University, New Brunswick, NJ USA

**Keywords:** Preschool, Early childhood policy, Head Start, Public schools, Developmentally appropriate practice

## Abstract

We investigated the extent to which practices considered developmentally appropriate and inappropriate varied by preschool program auspice (private, public school, and Head Start). Survey data from a 2010 national sample of 2,664 teachers of 4-year-olds provided teacher reports on the frequency of seven practices (e.g., offering children choices of play activities, using flashcards and math worksheets), approach to teaching subject matter, and time spent in whole group activities. More than 90 percent of teachers in all auspices reported high frequencies of some developmentally appropriate practices (DAP). Yet, private program teachers reported less DAP, more use of flashcards and worksheets, and more whole group time per day than teachers in the two public sectors. Some but not all differences by auspice could be explained by differences in teacher and classroom characteristics by auspice. In the context of other studies indicating little change in practice since 2010, our results suggest that increased public provision of preschool education does not lead to “academization.” We identify several other issues related to curriculum and “instruction” in preschool education requiring increased attention from researchers and policymakers.

## Introduction

In the United States, the early childhood field has long harbored fears that integration with the public schools and the standardization of early childhood programs through public policy would negatively influence practice (Elkind, 1969; Kagan, 1987; Fuller, 2008; Grubb, 1987; Institute of Medicine and National Research Council, 2015). These fears derive in part from concerns that educational practices inappropriate for younger children are pushed down from the primary grades but also from an emphasis on child outcomes and assessment in public accountability systems. Recently, claims of curriculum push down in the public schools have been put forward to explain why children who attended Tennessee’s state funded preschool program were found to perform worse on a broad range of measures of learning and development through 6th grade despite having performed better at kindergarten entry (Farran, 2022). This claim has been the basis for recommending that public funding be directed to private preschool programs rather than the public schools (Farran, 2022). Such a policy shift has important ramifications for democratic governance of preschool education, the role of the for-profit sector, and other issues as well as for program quality and the experiences young children have in publicly-funded preschool programs (Adamson et al., 2016; Brogaard & Helby Petersen, 2022; Richardson, 2022). In this paper we use data from a 2010 national survey of preschool teachers to investigate the historical association between public school auspice and the academization of preschool practices.

These and other concerns about the academization of early childhood education are important considerations for public policy not just in the United States but elsewhere (Gunnarsdottir, 2014; Hard & O'Gorman, 2007; Hayes, 2003; Justice et al., 2020; Nicolopoulou, 2010). What should the role of government be in funding, regulating, and operating preschool programs? Decisions about how to expand access to preschool education should be made carefully based on the best possible information. Public investments in preschool education can meaningfully enhance the well-being and development of young children as well as benefit society more broadly, and public policies should be designed to fulfill the promise with particular attention to avoiding harm (Barnett, 2011; Jancart et al., 2021). From this perspective it is vital to understand the extent to which public policies—including the choice between public and private auspices—may either support or undermine appropriate, child-centered practices or lead to “schoolifcation” of the preschool classroom.

This study uses data from a 2010 survey of preschool teachers in the United States to assess the frequency of practices associated with more academic, less play-based approaches and the extent to which these differ between public and private auspices and vary with other program features influenced by public policies. As fears that public schools would academize preschool education pre-date and post-date 2010, these data provide a basis for evaluating the extent to which such fears have been realized in the past. In addition, by investigating specific predictors of practice associated with differences in auspice we provide a basis for predicting impacts on practice of a shift toward public school provision in the current context. Although ours are the most recent national data available, we also discuss more current data on practice. Together with our results these provide a basis to inform current policy choices between privatization and public provision of preschool education.

We compare practices as reported by lead teachers across three auspices: state and locally funded public preschool, federally funded Head Start, and private (largely fee-based). These are the three major sectors operating preschool classrooms in the United States. State and locally funded public preschool programs are governed by state and local agencies but also are influenced by public policies that flow down from the federal level. There is substantial variation across states in both the agencies responsible and their policies, though structural features of preschool programs tend to be more uniform for classrooms housed in public schools (Friedman-Krauss et al., 2022a, 2022b). While Head Start operates under a single set of federal regulations, private fee-based providers operate independently, with government’s primary role to ensure health and safety. We conduct these analyses of differences by auspice with and without variables that measure teacher and classroom characteristics subject to public policies that as a result may also vary with auspice. For example, one might expect that one reason practices vary across auspice is because teacher characteristics vary systematically by auspice due to differences in compensation, qualifications requirements, and professional development (National Research Council, 2012). The primary goal of our study is to determine how much practice differs by auspice and other policy-related variables and whether teachers in public programs engage in more “academic” practices than those in private programs—but we also seek to improve understanding of the possible reasons for any differences in practice while recognizing the limits of our study as a basis for causal inferences.

### Context and previous research

Concerns with public school provision of preschool education have grown with increasing enrollment and a greater role for public schools. From 1970 to 2000, enrollment in preschool classrooms increased dramatically to just over half the population (National Center for Education Statistics, 2020). Preschool participation rates have changed modestly since, except for very recent declines due to the Covid-19 pandemic (National Center for Education Statistics, ). At the same time, the public sector has grown relative to the private sector with the public sector share growing substantially larger in the last two decades (McElrath et al., 2021). State-funded preschool accounts for much of the recent change as it expanded from 14 percent of 4-year-olds in 2001 to 34 percent in 2019 (Friedman-Krauss et al., 2021). Although most states have mixed delivery systems, in state-funded preschool programs public schools serve a majority of the children (Friedman-Krauss et al., 2022a, 2022b).

Historically, among the most frequently expressed concerns with the expansion of public education to serve younger children have been fears of the downward extension of the primary school curriculum and unrealistic expectations for children, the same fears that accompanied the earlier “absorption” of kindergarten into the public schools (Mitchell et al., 1989). For example, some have opined that “what was sold as a romantic and humanistic ‘garden of learning’ threatens to become just another grade level, committed to narrow cognitive skills and didactic teaching” (Fuller, 2008, p. xiii). McCabe and Sipple (2011) characterized the relationship between early childhood education and the public schools as “colliding worlds,” and cautioned that public schools engaged in preschool lack a full appreciation of the complexity of education for young children. Farran (2022) argued that prekindergarten in the public school harms children because it is unavoidably dominated by whole group instruction focused on basic skills of little long-term value and creates an aversive environment emphasizing negative control. This could decrease motivation and generate negative dispositions toward school that decrease later achievement.

Unfortunately, scant evidence is available to assess how well these assertions about public schools and preschool pedagogy comport with reality. Much of the research on variations in teaching practice and children’s experiences by auspice relies on restricted samples that are not widely generalizable (e.g., Fuligni et al., 2012; Stipek, et al., 1992; see studies cited by Bassok et al., 2016). The last nationally representative observational study of classroom quality was conducted in 2005 (Bassok et al., 2016; Coley et al., 2016). In that study, practice as measured by the Early Childhood Environmental Rating Scale differed relatively little by auspice, and the few differences favored Head Start and public schools over private programs (Bassok et al., 2016; Coley et al., 2016). More recent—but less representative—studies produced comparable results. One found public school and private preschools were more similar to each other than to kindergartens in the activities experienced by children, with much more free play, for example, while public school preschool teachers had more child-centered beliefs (Vitiello et al., 2020). Another found preschool children in public schools spent less time in whole group and transitions and more time in free choice activities than children in Head Start and private preschools (Nores et al., 2022). Denker & Atteberry (2024) provide a review of studies that measured time devoted to specific academic skills as well as to whole group and small group activities that shows both substantial variation by location but also remarkable consistency in patterns from years prior to our data to the present.

Associations between public school auspice or location and child-centered/teacher-directed practice may reflect the influences of other program characteristics associated with auspice or location. These include structural characteristics that can support process quality including child-centered and play-oriented practices (Cryer et al., 1999; NICHD Early Child Care Research Network, 2002). Program characteristics determined by auspice that might influence practice include teacher characteristics, operating schedule, and the population served. On average, public schools and Head Start offer better pay, have higher qualifications requirements, and are more likely to operate on part-day or school-day, school year schedules (Friedman-Krauss et al., 2022a, 2022b). Teacher race and ethnicity, experience, degree level, certification, and preservice and in-service training as well as the percentage of children from low-income families, length of day, staff-child ratio, and class size have been found to influence practice, though the evidence is mixed for all of them (Denny et al., 2012; Downer et al., 2016; Early et al., 2007; Pianta et al., 2005, 2009; Slot et al., 2015; Stipek & Byler, 1997; Tobin et al., 2013).

Although most concern for inappropriate practice and academization has focused on the public schools, similar fears have been raised about Head Start. For example, some have worried that increased emphasis on accountability for school readiness in Head Start led to a push down of primary school type practices within Head Start (Walter & Lippard, 2017). Research with nationally representative data on Head Start teacher-reported practices suggests complex changes over time, but these are difficult to interpret (Markowitz & Ansari, 2020). The questions teachers were asked are ambiguous regarding the appropriateness of practice. More specifically, teachers were asked how much time children spent in “teacher-directed” whole group, small group and individual activities. Teacher-directed activities are not synonymous with direct instruction, focused on rote learning of discrete skills or facts, or devoid of child initiation and choice. Nor is teacher-initiated intentional teaching—particularly in small group or one-on-one—necessarily inappropriate or academic (Dickinson, 2002). Nationally representative observation data for Head Start indicate that quality improved over time rather than declined (Office of Head Start, 2016).

### Theoretical framework

The theoretical basis for claims that public schools will tend to increase overly academic, narrowly skills-focused practice is unclear in the literature. Claims regarding the academization of preschool education largely appear to stem from concerns that educational standards and integration with the public education lead to push down of methods from the elementary school (Bagnato & Ho, 2006; Elkind, 1969). Although this might occur simply as a result of preschool being incorporated into a larger structure governed and administered by a system dominated by elementary and secondary professionals, a major source of concern is that public accountability systems’ emphases on academic outcomes would drive teachers away from play-based approaches and child-centered pedagogy (Cade et al., 2022; Fuller, 2008). It is reasonable to suppose that public school district and building leadership would have less early childhood specific training. This need not be true for preschool teachers and early childhood supervisors in the public schools, however. All states have early learning standards (ELS) developed by early childhood experts (as does Head Start) and aligned with national education goals. Some have argued that ELS introduce a framework that could lead to inappropriate practices, but these claims have not been empirically tested (Merill et al., 2020).

A counter-argument can be made that public schools and Head Start might make greater use of developmentally appropriate practices because they have stronger teacher preparation and professional development than private providers. Epstein (1999) found that formal education levels of teachers predicted quality in public schools while in-service professional development predicted quality in Head Start. A meta-analysis by Manning et al. (2019) found higher teacher qualifications associated with higher quality. Another meta-analysis (Egert et al., 2020) found that in-service training improves teacher–child interactions.

To address the question of negative effects on practice we must define theoretically and operationally what constitutes good or appropriate practice. As Hatch (2019) details, developmental theory provides little guidance regarding the content of preschool curriculum and emphases the methods or “how” of early childhood education. This is evident in statements of developmentally appropriate practice (DAP) (Hatch, 2019; National Association for the Education of Young Children, 2020). Confusion between “what” and “how” is one source of false dichotomies in the field between play (how) and academics (what), and misconceptions of a necessary alignment between what and how may explain much of the concern about academization (Clements & Sarama, 2014; Hirsh-Pasek & Golinkoff, 2011). However, differences in theoretical perspective (e.g., Piaget v. Vygotsky) also lead to different views about the importance of specifying or even attending to what is taught, children’s capabilities, and the teacher’s role in the classroom (Hatch, 2019). That said, our analysis of the critiques of public school preschool pedagogy is that public preschool prograns focus how children are taught rather than what they are taught.

These perspectives lead us to focus our study on specific approaches cited by those concerned with academization and on specific types of activities that are key elements of DAP (Bredekamp & Copple, 1997). These focus on how preschool are taught rather than what they are taught. These include free choice, exploration, hands on activities, the use of flash cards and worksheets, integration of subject matter, and the amount of time spent in whole group and small group activities. As we discuss below, we recognize that visions of DAP are complex and vary within the field as well as the fact that whole group activities are not necessarily inappropriately didactic. Nevertheless, the measures of practice that we examine broadly represent the specific practices cited in concerns about academization (Cade et al., 2022; Elkind, 1969; Grubb, 1987). More specifically, we assessed DAP with five questions regarding practice strongly associated with the principles of DAP and two questions regarding specific practices that are typically viewed as exemplars of inappropriate practice (see for example Charlesworth et al., 1991).

## Methods

### Participants

Data are from a survey of 2,664 preschool teachers from across the United States in Fall 2010. Our target sample size was at least 2400 (800 per auspice) to provide a 95% confidence interval for percentages of plus or minus 2 percent (and 4 percent for each auspice). We sampled randomly from a list of approximately 5,000 preschool centers stratified by auspice. The list was obtained from a commercial provider of lists for marketing purposes. At the time of the survey there was no source for a complete list of preschool centers or teachers that included Head Start, public schools, and fee-based private providers. Each institution was called and asked for a list of teachers of 4-year-olds and then one teacher from each center was randomly selected and asked to complete the survey. If that teacher was unavailable (for example, due to illness), another was selected at random from that center’s list. Teachers could have had mixed age groups or taught multiple sessions with different ages; it was only necessary that some of their students be 4-year-olds.

### Survey procedures and measures

Data were collected by trained interviewers at a commercial firm using a fully scripted protocol in a computer-aided telephone interview that included obtaining informed consent. The interview took about 12 min to complete and was administered in one session. Teachers received $10 for completing the interview. Prior to data collection, the protocol was field tested and revised based on feedback from preschool teachers and early childhood education experts. Initial interviews were randomly monitored live for potential unanticipated difficulties, but none were detected. Analyses for this study were conducted with a datafile containing no identifying information on respondents.

The survey obtained self-reported information on preschool teachers’ practices as well as personal (e.g., gender and race/ethnicity) and professional (e.g., education and training, experience, salary, and benefits) characteristics. It also verified the auspices of the classrooms in which they worked. This allowed us to link teacher reports on practices to program auspice and to teacher and classroom characteristics.

Teachers reported on five activities we view as indicators of child-initiated, play-oriented DAP. They were asked to rate the frequency of these activities on the following Likert scale: never, rarely (1 time per month), sometimes (1 time per week), regularly (2 to 4 times per week), very often (daily or nearly every day). The five questions for these activities are below.

- How often do children in your preschool classrooms regularly have the opportunity to build or play with blocks?
- How often do children in your classroom have the chance to select from a variety of learning areas and projects?
- How often do the children in your class have the opportunity to explore science materials, such as animals, plants, wheels, or gears?
- How often do your students see, hear, or experience their own culture and language?
- How often do children in your classroom have opportunities to engage in their own choice of play activities?

Teachers also answered two other questions that we view as indicators of teacher-centered, didactic practices. These relate to flashcards and worksheets which, in addition to being more teacher-centered, limit opportunities for meaningful individual teacher–child conversations (Hamre, 2014). Teachers used the same rating scale for frequency to respond to these questions below.

- How often do you use flashcards containing the alphabet or sight words as a way to increase children’s learning?
- How often do you use worksheets to help children learn math?

In addition, we asked two other questions that can provide insights into the teacher’s orientation toward practice and what might be called “schoolification.”

- Do you usually teach academic subjects separately, or integrate multiple subjects at the same time?
- On average, how many total minutes per day do you usually spend teaching the whole class all at once as a way to help children learn certain skills or topics?

These last two questions ask about the amount of time in whole group activities and integration of subject matter throughout the day both of which help differentiate child-centered, whole-child approaches from more didactic methods common to the primary grades for older children (Kostelnik et al., 2019). The responses to these last two questions require careful consideration. For example, a whole group activity might be full group dramatic play or singing, and the size of the whole group might be relatively small in some classrooms. Nevertheless, we think it is reasonable that more time in whole group activities and less integration of subject areas suggest a less child-centered, individualized approach.

We defined auspice as sponsorship by public school (state or school district administered), Head Start, or private. We coded auspice based on each teacher’s individual classroom recognizing that centers can have multiple auspices and funding sources. This was not always easily discerned or unambiguous. Initial data review indicated some potential problems with the coding of auspice by the commercial interviewers. We conducted follow-up calls with center directors (as the most knowledgeable person about administration and funding) using graduate assistants knowledgeable about private provider and Head Start participation in state-funded preschool to clarify auspice. Although we refer to them as “public school” programs, not all state-funded preschool programs are administered by state education agencies, and these programs may blend federal, state, and local education funding with Head Start and child care funds (Friedman-Krauss et al., 2022a, 2022b).

We constructed an alternative measure of auspice that classified teachers based solely on location that allowed us to assess the sensitivity of our results to how auspice is conceptualized and classified. For this measure, any teacher in a classroom located in a public school was coded as “public school” for auspice even if they were reported to be in Head Start or a private program. Any teacher reported as located in a Head Start and not in a public school was coded as Head Start. Everyone else was coded as private (a small number on military bases or at colleges and universities are publicly funded but they are neither public school nor Head Start). Although this alternative measure does not correspond as closely to administrative auspice, the classification of classrooms by location was less ambiguous. Moreover, this measure allows us to consider location per se as an influence on practice. It could be argued that location in a public school and proximity to primary school classrooms has a stronger influence on practice than funding stream and administrative authority.

### Research questions

This study addresses four broad research questions:

1. How do teachers’ reported practices vary by auspice?
2. How much variation is there in teachers’ reported practices within each auspice?
3. To what extent do direct associations between auspice and teachers’ reported practices persist after taking into account teacher and other program or classroom characteristics that vary by auspice.
4. What other teacher and program characteristics are associated with teachers’ reported practices that might also be influenced by public policy?

### Data analyses

We first conducted descriptive analyses to obtain an overview of the sample and assess the distributions of each variable including the number of missing cases. None of the variables have even 0.2 percent missing, and as a result we do not employ procedures to impute missing data. Next we conducted analyses to examine the associations of auspice and practice. Seven of our nine measures of practice use Likert scales. Given the controversies regarding analysis of Likert scales we conducted nonparametric and parametric analyses for these variables (Mircioiu & Atkinson, 2017). Analyses of the association of the Likert scale responses with auspice alone were MANOVA and Chi-square. The MANOVA assesses the association with auspice for an entire vector of practice measures rather than for each of the seven practices separately. We employed logistic regression to examine the association between auspice and the other two measures of practice—integration of subject matter and the amount of whole group time. For the logistic analyses, we categorized integration of subject matter as integrated versus all others, and we categorized whole group time into two levels, up to 30 min versus more than 30 min. Finally, we present graphical analyses by auspice for all of the practice measures.

For the seven practices measured on Likert scales, the MANOVA and Chi-square analyses were followed-up with individual regression analyses for each practice. We conducted these regressions first with auspice alone and then with the addition of teacher and classroom characteristic variables. These multivariate regressions provide insights into the extent to which variations by auspice may be related to teacher and classroom characteristics influenced by auspice. All regression analyses were repeated with the alternative measure of auspice based on location to assess the sensitivity of results to how auspice was defined. Auspice was represented by binary variables for public school and Head Start with private programs as the reference group. Teacher and classroom variables included were: binary variables for race/ethnicity (Black, Hispanic, and Other, with White-Non-Hispanic as the reference group), teacher years of experience, teacher education level, early childhood certification (yes = 1; no = 0), other teaching certification (yes = 1; no = 0), teacher language (1 = English only, 0 = other), teaching a single session per day or multiple sessions (one session = 1; other = 0), number of other adults in the classroom during a typical day, enrollment per session taught, average family income of children (low income = 1; other = 0), and region (Northeast is the reference group, binary variables for Midwest, West, South).

## Results

The total sample of 2,664 teachers consisted of 1,017 (38%) teachers in private providers, 829 (31%) teachers in public school programs (state-funded or school district) and 818 (31%) teachers in Head Start. Table 1 presents descriptive statistics by auspice for teacher and program characteristics. Teacher and student characteristics varied by auspice as might be expected given differences by auspice in standards and regulations regarding teacher qualifications and in the eligibility criteria (e.g., Head Start and many public preschool programs limit enrollment to children in low-income families, and some public programs are designed for children with special needs). As noted earlier, we also calculated an alternative auspice measure defined solely by location. Using this measure, the sample is comprised of 886 (34%) private provider teachers, 972 (36%) public school teachers, and 777 (29%) Head Start teachers for at total sample of 2635 (29 did not report location).

**Table 1:** Descriptive Statistics

		Private		Public		Head Start	
		N	%	N	%	N	%
Total		1017		829		818	
Race/Ethnicity	Black	165	16.2%	88	10.6%	180	22%
	Hispanic	97	9.5%	97	11.7%	112	13.7%
	White	685	67.4%	601	72.5%	480	58.7%
	Asian	34	3.3%	19	2.3%	11	1.3%
	Native American	9	0.9%	6	0.7%	17	2.1%
	Other	26	2.6%	18	2.2%	16	2.0%
	Refused	1	0.1%	–	–	2	0.2%
Teaching experience	Less than 5 years	526	51.7%	312	37.6%	338	41.3%
	5 to 10 years	233	22.9%	230	27.7%	217	26.5%
	10 to 20 years	191	18.8%	229	27.6%	200	24.4%
	20 years and above	67	6.6%	56	6.8%	62	7.6%
	Don't Know	–	–	2	0.2%	1	0.1%
Education level	Less than high school	5	0.5%	1	0.1%	–	–
	High school diploma /GED/Some College	239	23.5%	34	4.1%	43	5.3%
	Currently enrolled in an AA/AAS/BA	114	11.2%	24	2.9%	70	8.6%
	Associates/AA/AAS	179	17.6%	51	6.2%	179	21.9%
	BA or above	478	47.0%	717	86.5%	526	64.3%
	Don’t know	2	0.2%	2	0.2%	–	–
Session	One session	842	82.8%	557	67.2%	673	82.3%
	Two sessions	126	12.4%	250	30.2%	142	17.4%
	Other	49	4.8%	22	2.7%	3	0.3%
Language	English Only	789	77.6%	661	79.7%	629	76.9%
	Spanish	137	13.5%	121	14.6%	142	17.4%
	Other	91	8.9%	47	5.7%	47	5.7%
Early Childhood Certification	Yes	149	14.7%	427	51.5%	218	26.7%
	No	868	85.3%	402	48.5%	600	73.3%
Other Certification	Yes	134	13.2%	195	23.5%	120	14.7%
	No	883	86.8%	634	76.5%	698	85.3%
Region	Northeast	133	13.1%	159	19.2%	174	21.3%
	Midwest	328	32.3%	179	21.6%	192	23.5%
	South	327	32.2%	317	38.2%	260	31.8%
	West	229	22.5%	174	21.0%	192	23.5%
Family Income	Low income	183	18.0%	470	56.7%	677	82.8%
of children	Lower-middle income	90	8.8%	85	10.3%	47	5.7%
	Middle income	506	49.8%	201	24.2%	60	7.3%
	Upper-Middle income	83	8.2%	14	1.7%	5	0.6%
	Upper income	88	8.7%	6	0.7%	3	0.4%
	Mixed	27	2.7%	26	3.1%	9	1.1%
	Don't Know	39	3.8%	23	2.80%	17	2.1%
	Refused	1	0.1%	4	0.50%	–	–

	Private		Public		Head Start	
	Mean	SD	Mean	SD	Mean	SD
Enrollment per Session	16.61	9.58	17.58	7.00	17.48	5.89
Number of Other Staff in Room	1.23	1.01	1.63	1.05	1.57	0.83
	Mean	%	Mean	%	Mean	%
IEP (number of children in class with IEP)	2.68	6.5%	5.32	22.7%	4.01	15.4%

		Private		Public		Head Start	
		N	%	N	%	N	%
Curriculum Integration	Integration of multiple subject matter	668	66.1%	708	85.4%	659	81.1%
	Other	343	33.7%	121	14.6%	154	18.9%
Daily Time in Whole Group	Up to 30 min	402	39.8%	382	46.4%	470	57.7%
	More than 30 min	609	60.2%	441	53.6%	344	42.3%

### Association of practices with auspice

**Fig. 1:**
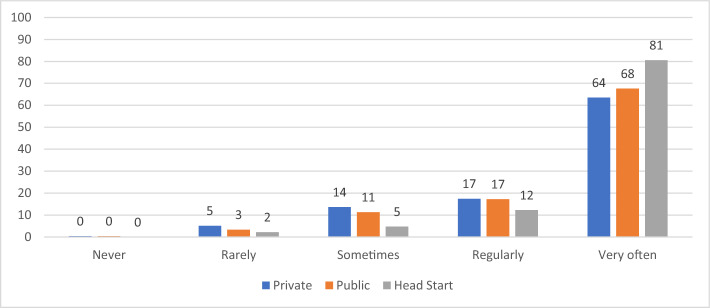
Frequency of Opportunities to Explore Science Materials by Auspice

**Fig. 2:**
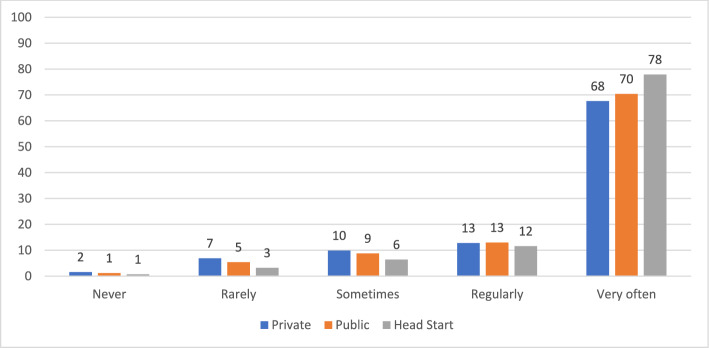
Frequency of Opportunities to Experience Child’s Own Culture and Language by Auspice

**Fig. 3:**
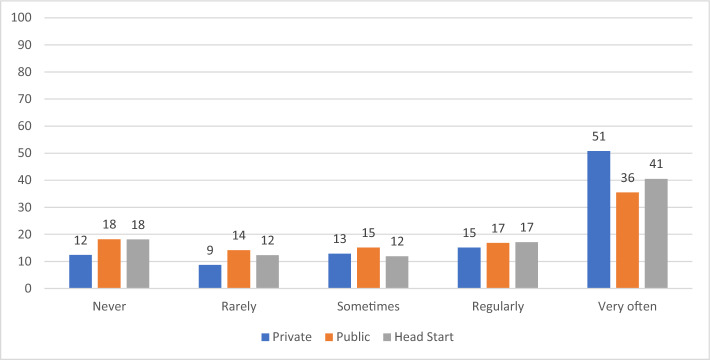
Frequency of Use Flashcards by Auspice

**Fig. 4:**
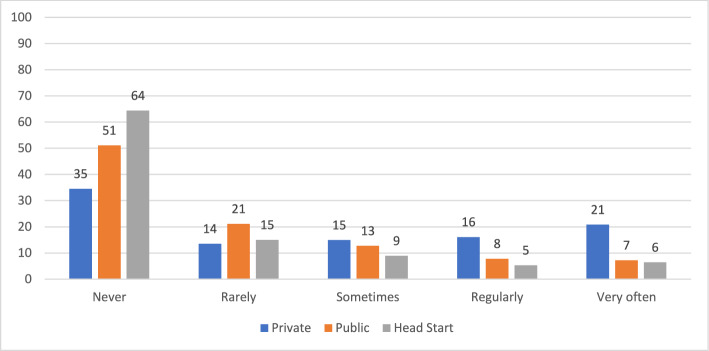
Frequency of Use Worksheets by Auspice

We find clear differences in practices by auspice. As shown in Table 2, we found statistically significant (χ2, *p* < 0.01) differences in the distributions of all seven Likert-scale measures of practice by auspice. For three of the measures more than 90 percent of the teachers in each auspice reported the highest frequency, so the differences by auspice were quite small. For the other variables, the distributions varied considerably more by auspice. Among the three auspices, private providers always had the lowest frequency of more play-based, centered approaches and the highest frequency of more academic practices. Figures 1, 2, 3, 4 display results graphically for the practices that varied most by auspice.

**Table 2:** Teacher Reported Practices by Auspice: Chi-square Tests

Measures	Auspice		Frequency					Chi square tests	
			Never	Rarely/ Once a month	Sometimes/ Once a week	Regularly/2–4 times per week	Very often / daily or almost everyday		
Play with blocks	Private	N (%)	5 (0.5%)	2 (0.2%)	8 (0.8%)	64 (6.3%)	938 (92.2%)		
	Public	N (%)	0	1 (0.1%)	8 (1.0%)	40 (4.8%)	779 (94.1%)	χ^2^ (8) = 23.01, *p* < .01	
	Head start	N (%)	0	1 (0.1%)	3 (0.4%)	23 (2.8%)	791 (96.7%)		
Variety of learning areas	Private	N (%)	2 (0.2%)	4 (0.4%)	10 (1.0%)	49 (4.8%)	952 (93.6%)		
	Public	N (%)	0 (0%)	2 (0.2%)	6 (0.7%)	21 (2.5%)	800 (96.5%)	χ^2^ (8) = 27.83, *p* < .001	
	Head start	N (%)	2 (0.2%)	0	0	15 (1.8%)	801 (97.9%)		
Explore science materials	Private	N (%)	4 (0.4%)	52 (5.1%)	138 (13.6%)	177 (17.4%)	646 (63.5%)		
	Public	N (%)	3 (0.4%)	28 (3.4%)	94 (11.4%)	143 (17.3%)	560 (67.6%)	χ^2^ (8) = 78.78, *p* < .001	
	Head Start	N (%)	0	18 (2.2%)	39 (4.8%)	101 (12.4%)	659 (80.7%)		
Experience own culture/language	Private	N (%)	16 (1.6%)	70 (7.0%)	101 (10.0%)	130 (12.9%)	688 (68.5%)		
	Public	N (%)	10 (1.2%)	45 (5.5%)	73 (8.9%)	108 (13.2%)	584 (71.2%)	χ^2^ (8) = 28.94, *p* < .001	
	Head Start	N (%)	6 (0.7%)	26 (3.2%)	52 (6.4%)	95 (11.6%)	637 (78.1%)		
Choose own play activities	Private	N (%)	1 (0.1%)	2 (0.2%)	19 (1.9%)	49 (4.8%)	945 (93%)		
	Public	N (%)	0	0	9 (1.1%)	28 (3.4%)	792 (95.5%)	χ^2^ (8) = 24.44, *p* < .01	
	Head Start	N (%)	0	1 (0.1%)	0	23 (2.8%)	794 (97.1%)		
Flashcards	Private	N (%)	126 (12.4%)	88 (8.7%)	131 (12.9%)	154 (15.2%)	517 (50.9%)		
	Public	N (%)	151 (18.3%)	118 (14.3%)	125 (15.1%)	139 (16.8%)	294 (35.6%)	χ^2^ (8) = 57.58, *p* < .001	
	Head Start	N (%)	148 (18.1%)	101 (12.4%)	97 (11.9%)	140 (17.1%)	331 (40.5%)		
Worksheets	Private	N (%)	351 (34.5%)	137 (13.5%)	152 (15%)	164 (16.1%)	212 (20.9%)		
	Public	N (%)	424 (51.1%)	175 (21.1%)	105 (12.7%)	65 (7.8%)	60 (7.2%)	χ^2^ (8) = 276.5, *p* < .001	
	Head Start	N (%)	527 (64.4%)	123 (15%)	73 (8.9%)	43 (5.3%)	52 (6.4%)

Table 3 reports the results of MANOVA, revealing significant differences in outcomes across child care settings (*p* < 0.001), thereby confirming disparities in educational practices among the various auspices. These findings were further examined using univariate ANOVAs with a Bonferroni correction set at *p* < 0.007, which confirmed significant differences for each measure. Tukey's HSD post-hoc testsprovided additional insights into the nature of these differences. Similarly, private programs provided fewer opportunities for children to engage in self-directed play activities compared to public schools (*p* < .05) and Head Start (*p* < .001). The availability of science-related materials was also lower in private programs than in both public schools (*p* < .05) and Head Start (*p* < .001). Furthermore, Head Start demonstrated a notable advantage over public schools in providing access to science materials (*p *< .001). Cultural exposure was another area where Head Start programs outperformed both private and public settings, providing more opportunities for children to experience their own culture and language, against private (*p* < .001) and public settings (*p* < .01).

**Table 3:** Teacher Reported Practices by Auspice: Multivariate Analysis of Variance and One-Way Analysis of Variance

Measure	Auspice									
	Private^a^		Public^b^		Head Start^c^		ANOVA		Tukey	
	Mean	SD	Mean	SD	Mean	SD	F	*p*	Comparison	Mean difference
Play with blocks	4.89	0.43	4.93	0.32	4.96	0.23	8.78	< .001	a-c	−0.068***
Variety of learning areas	4.91	0.38	4.95	0.27	4.97	0.24	8.79	< .001	a-b	−0.039*
									a-c	−0.060***
Explore science materials	4.39	0.93	4.48	0.85	4.72	0.65	37.91	< .001	a-b	−0.098*
									a-c	−0.334***
									b-c	−0.236***
Experience own culture/language	4.40	1.03	4.47	0.95	4.63	0.80	14.34	< .001	a-c	−0.235***
									b-c	−0.157**
Choose own play activities	4.90	0.39	4.94	0.27	4.97	0.20	10.74	< .001	a-b	−0.042*
									a-c	−0.065***
Flashcards	3.84	1.43	3.37	1.53	3.50	1.55	24.70	< .001	a-b	0.473***
									a-c	0.346***
Worksheets	2.76	1.57	1.99	1.27	1.73	1.20	139.37	< .001	a-b	0.764***
									a-c	1.025***
									b-c	0.261*

Wilks' Lambda = 0.878, F (14, 5252) = 25.15, *p* < 0.001

*p < 0.05, ** p < 0.01, ***p < 0.001

 Significant differences were also observed in the use of instructional materials such as math worksheets and flashcards. Private providers reported more frequent use of these tools compared to both public schools and Head Start (*p* < .001). Public school teachers, in turn, reported greater usage of math worksheets compared to their counterparts in Head Start (*p* < .05). Looking at both Tables 2 and 3, the largest differences in practice by auspice were for worksheets and flashcards. Most private provider teachers reported that they used math worksheets at least sometimes and that they used flashcards very often. By contrast, most Head Start and public school teachers reported never using math worksheets and using flashcards less than very often.

**Fig. 5:**
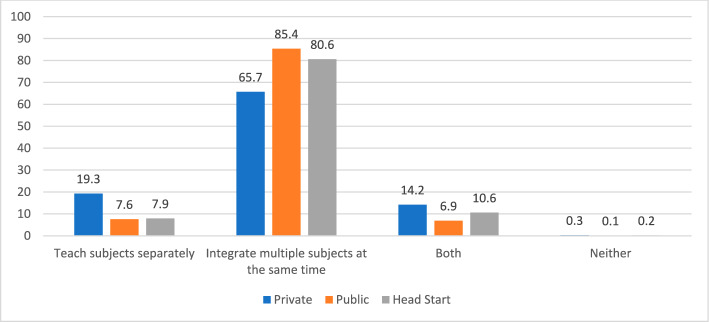
Approaches to Curriculum by Auspice: Teaching Subjects Separately or Integrating

**Fig. 6:**
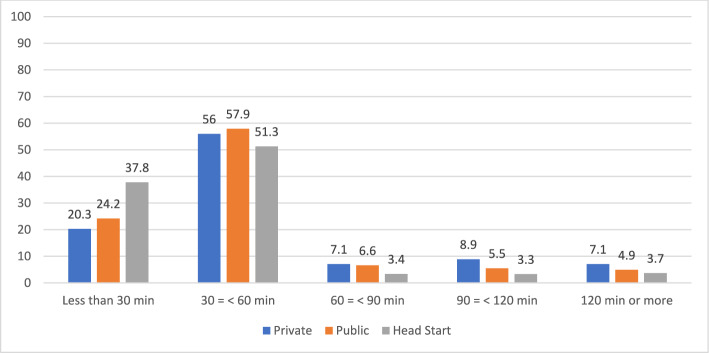
Daily Time in Whole Group by Auspice

Results for the other measures of practice—curriculum integration and whole group time—are consistent with those presented above. Distributions by auspice are graphed in Figs. 5 and 6. As seen in Table 4, logistic regression analysis revealed that auspice was a significant predictor of curriculum integration (*p* < . 001). Compared to private provider teachers, public school teachers were almost three times as likely (odds ratio = 2.98), while Head Start teachers were over twice as likely (odds ratio = 2.23), to integrate subject matter. After accounting for teacher and program covariates in the logistic regression model, the association remained statistically significant (*p* < 0.001), although the effect was slightly reduced (odds ratio: Public school: 1.82; Head Start 1.74). The relationship was more pronounced and statistically significant when the auspice was defined based on location.

**Table 4:** Effects of Auspice on Practice: Logistic Regression Estimates with and without Controls for Teacher and Child Characteristics with Auspice

	(1)	(2)	(3)	(4)	(5)^a^	(6)^a^	(7)^a^	(8)^a^
	Integration of curriculum	Integration of curriculum	Daily time in whole group	Daily time in whole group	Integration of curriculum	Integration of curriculum	Daily time in whole group	Daily time in whole group
	b/se	b/se	b/se	b/se	b/se	b/se	b/se	b/se
Public School	1.092^***^	0.596^***^	−0.270^**^	−0.136	1.156^***^	0.699^***^	−0.238^*^	−0.071
	(0.12)	(0.14)	(0.10)	(0.12)	(0.11)	(0.14)	(0.09)	(0.12)
Head Start	0.802^***^	0.555^***^	−0.727^***^	−0.629^***^	0.854^***^	0.676^***^	−0.791^***^	−0.700^***^
	(0.11)	(0.14)	(0.10)	(0.12)	(0.11)	(0.14)	(0.10)	(0.12)
Black		−0.540^***^		−0.040		−0.542^***^		−0.020
		(0.13)		(0.12)		(0.13)		(0.12)
Hispanic		−0.398^*^		−0.342^*^		−0.424^*^		−0.319
		(0.19)		(0.17)		(0.19)		(0.17)
Other Race		−0.426^*^		−0.241		−0.467^*^		−0.219
		(0.21)		(0.18)		(0.21)		(0.18)
Experience		0.012		−0.021^***^		0.012		−0.022^***^
		(0.01)		(0.01)		(0.01)		(0.01)
Education		0.123^***^		−0.051		0.123^***^		−0.056^*^
		(0.03)		(0.03)		(0.03)		(0.03)
Number of Staff		0.017		0.023		0.004		0.029
		(0.05)		(0.04)		(0.05)		(0.04)
One session		−0.323^*^		0.356^***^		−0.316^*^		0.372^***^
		(0.14)		(0.10)		(0.14)		(0.10)
Enrollment/session		0.004		−0.005		0.004		−0.006
		(0.01)		(0.01)		(0.01)		(0.01)
IEP		0.257		−0.057		0.241		−0.086
		(0.26)		(0.19)		(0.26)		(0.19)
Low Income		0.219		−0.056		0.177		−0.038
		(0.12)		(0.10)		(0.12)		(0.10)
English only		0.214		−0.317^*^		0.202		−0.315^*^
		(0.14)		(0.13)		(0.14)		(0.13)
EC certification		0.223		0.111		0.209		0.065
		(0.14)		(0.11)		(0.14)		(0.11)
Other certification		0.233		0.224		0.221		0.192
		(0.16)		(0.13)		(0.16)		(0.13)
Midwest		0.186		0.100		0.197		0.093
		(0.16)		(0.13)		(0.17)		(0.13)
South		−0.137		0.328^**^		−0.127		0.307^*^
		(0.15)		(0.12)		(0.15)		(0.12)
West		−0.221		0.161		−0.193		0.128
		(0.17)		(0.14)		(0.17)		(0.14)
Constant	0.662^***^	0.008	0.418^***^	0.716^**^	0.575^***^	−0.050	0.431^***^	0.770^**^
	(0.07)	(0.30)	(0.06)	(0.25)	(0.07)	(0.30)	(0.07)	(0.26)
*N*	2634	2634	2629	2629	2634	2634	2629	2629
*OR Public School*	2.983	1.815	0.763	0.872	3.175	2.009	0.788	0.931
*OR Head Start*	2.227	1.744	0.483	0.533	2.348	1.967	0.454	0.497

*p < 0.05, **p < 0.01, ***p <0 .001

^a^Used Auspice by location

*OR* Odds Ratio

Logistic regression analysis found a significant association between auspice and the use of whole group time. Public school teachers had a 24% lower likelihood (odds ratio = 0.76) of using whole group time, while Head Start teachers had 52% lower likelihood (odds ratio = 0.48) compared to private provider teachers. When the definition of auspice was based on location, the findings remained consistent.

### Association of practices with auspice controlling for teacher and program characteristics

Regression analysis for each of seven instructional practices measured on Likert scales found that the addition of teacher and program characteristics reduced but did not eliminate the associations between auspice and practice as shown in Tables 5 and 6. Head Start continued to be a significant predictor for five of the seven practices: use of flashcards and worksheets, exposure to the child’s culture and home language, and choice of play activities and science materials. Focusing on auspice per se, public school remained a significant predictor only for worksheets. However, when auspice was defined by location, public school also remained significantly associated with more frequent exposure to the child’s culture and home language, and choice of science materials. No regression analysis indicated that private provider teachers reported more use of play-based, child-centered practice than public school or Head Start teachers. The addition of teacher and program characteristics to the regression model increased adjusted *R*^*2*^ in addition to reducing the size of the coefficients for public school and Head Start auspices.

**Table 5:** Effects of Auspice on Practice: Regression Estimates with and without Controls for Teacher and Child Characteristics

	(1)	(2)	(3)	(4)	(5)	(6)	(7)	(8)	(9)	(10)	(11)	(12)	(13)	(14)
	Blocks	Blocks	Learning Area	Learning Area	Science	Science	Culture	Culture	Choice time	Choice time	Flash cards	Flash cards	Worksheets	Worksheets
	b/se	b/se	b/se	b/se	b/se	b/se	b/se	b/se	b/se	b/se	b/se	b/se	b/se	b/se
Public School	0.033^*^	0.023	0.040^**^	0.013	0.099^*^	0.075	0.081	0.066	0.040^**^	0.009	−0.451^***^	−0.120	−0.761^***^	−0.407^***^
	(0.02)	(0.02)	(0.01)	(0.02)	(0.04)	(0.05)	(0.04)	(0.05)	(0.01)	(0.02)	(0.07)	(0.08)	(0.06)	(0.08)
Head Start	0.066^***^	0.037	0.060^***^	0.030	0.330^***^	0.250^***^	0.237^***^	0.124^*^	0.064^***^	0.034^*^	−0.334^***^	−0.184^*^	−1.009^***^	−0.758^***^
	(0.02)	(0.02)	(0.01)	(0.02)	(0.04)	(0.05)	(0.04)	(0.05)	(0.01)	(0.02)	(0.07)	(0.08)	(0.06)	(0.08)
Black		−0.051^**^		−0.025		−0.059		0.056		−0.032		0.817^***^		0.584^***^
		(0.02)		(0.02)		(0.05)		(0.05)		(0.02)		(0.08)		(0.07)
Hispanic		0.044		−0.018		−0.135^*^		0.059		0.001		0.461^***^		0.268^*^
		(0.03)		(0.03)		(0.07)		(0.08)		(0.02)		(0.12)		(0.11)
Other Race		−0.021		−0.037		−0.194^**^		−0.096		−0.017		0.390^**^		0.332^**^
		(0.03)		(0.03)		(0.07)		(0.08)		(0.03)		(0.13)		(0.12)
Experience		−0.001		0.001		0.002		−0.002		0.001		−0.013^**^		−0.004
		(0.00)		(0.00)		(0.00)		(0.00)		(0.00)		(0.00)		(0.00)
Education		0.006		0.007		−0.005		0.018		0.013^***^		−0.101^***^		−0.073^***^
		(0.00)		(0.00)		(0.01)		(0.01)		(0.00)		(0.02)		(0.02)
Number of Staff		0.018^*^		0.004		0.045^**^		0.003		0.011		−0.072^*^		−0.079^**^
		(0.01)		(0.01)		(0.02)		(0.02)		(0.01)		(0.03)		(0.03)
One session		0.005		0.016		0.080		0.062		0.010		0.035		−0.030
		(0.02)		(0.02)		(0.04)		(0.05)		(0.02)		(0.07)		(0.07)
Enrollment/session		0.002^*^		0.001		0.005^*^		0.003		0.001		0.006		−0.002
		(0.00)		(0.00)		(0.00)		(0.00)		(0.00)		(0.00)		(0.00)
IEP		−0.098^**^		0.014		−0.228^**^		−0.228^**^		−0.001		0.051		0.235
		(0.03)		(0.03)		(0.08)		(0.09)		(0.03)		(0.13)		(0.12)
Low Income		0.048^**^		0.032^*^		0.139^***^		0.200^***^		0.027		−0.137^*^		−0.292^***^
		(0.02)		(0.01)		(0.04)		(0.04)		(0.01)		(0.07)		(0.06)
English only		0.037		0.021		−0.036		−0.076		0.013		−0.319^***^		−0.384^***^
		(0.02)		(0.02)		(0.05)		(0.06)		(0.02)		(0.09)		(0.08)
EC certification		−0.034		−0.003		−0.006		−0.104^*^		−0.023		−0.086		−0.121
		(0.02)		(0.02)		(0.05)		(0.05)		(0.02)		(0.08)		(0.07)
Other certification		−0.044^*^		−0.022		−0.047		−0.034		−0.010		0.038		−0.112
		(0.02)		(0.02)		(0.05)		(0.06)		(0.02)		(0.09)		(0.08)
Midwest		0.007		0.001		−0.009		0.107		0.017		−0.134		0.084
		(0.02)		(0.02)		(0.05)		(0.06)		(0.02)		(0.09)		(0.08)
South		0.018		−0.018		0.086		0.082		−0.009		0.165		0.047
		(0.02)		(0.02)		(0.05)		(0.06)		(0.02)		(0.08)		(0.08)
West		−0.044^*^		−0.013		−0.026		0.004		0.006		−0.007		0.218^*^
		(0.02)		(0.02)		(0.05)		(0.06)		(0.02)		(0.09)		(0.09)
Constant	4.895^***^	4.792^***^	4.912^***^	4.828^***^	4.384^***^	4.238^***^	4.393^***^	4.194^***^	4.904^***^	4.781^***^	3.832^***^	4.513^***^	2.753^***^	3.486^***^
	(0.01)	(0.04)	(0.01)	(0.04)	(0.03)	(0.10)	(0.03)	(0.12)	(0.01)	(0.04)	(0.05)	(0.17)	(0.04)	(0.16)
*N*	2644	2644	2645	2645	2643	2643	2622	2622	2644	2644	2641	2641	2644	2644
*R-squared *_*adj*_	0.006	0.021	0.006	0.010	0.026	0.043	0.010	0.025	0.007	0.015	0.016	0.117	0.093	0.162

*p < 0.05, **p < 0.01, ***p <0 .001

**Table 6:** Effects of Auspice on Practice: Regression Estimates with and without Controls for Teacher and Child Characteristics with Auspice Measured by Location

	(1)	(2)	(3)	(4)	(5)	(6)	(7)	(8)	(9)	(10)	(11)	(12)	(13)	(14)
	Blocks	Blocks	Learning Area	Learning Area	Science	Science	Culture	Culture	Choice time	Choice time	Flash cards	Flash cards	Worksheets	Worksheets
	b/se	b/se	b/se	b/se	b/se	b/se	b/se	b/se	b/se	b/se	b/se	b/se	b/se	b/se
Public School loc	0.038^*^	0.027	0.035^*^	0.006	0.119^**^	0.098^*^	0.121^**^	0.115^*^	0.045^**^	0.015	−0.436^***^	−0.089	−0.828^***^	−0.482^***^
	(0.02)	(0.02)	(0.01)	(0.02)	(0.04)	(0.05)	(0.04)	(0.05)	(0.01)	(0.02)	(0.07)	(0.08)	(0.06)	(0.07)
Head Start loc	0.065^***^	0.034	0.063^***^	0.033	0.365^***^	0.294^***^	0.262^***^	0.154^**^	0.070^***^	0.044^*^	−0.333^***^	−0.203^*^	−1.073^***^	−0.852^***^
	(0.02)	(0.02)	(0.02)	(0.02)	(0.04)	(0.05)	(0.05)	(0.06)	(0.01)	(0.02)	(0.07)	(0.09)	(0.07)	(0.08)
Black		−0.050^**^		−0.026		−0.064		0.056		−0.033		0.822^***^		0.591^***^
		(0.02)		(0.02)		(0.05)		(0.05)		(0.02)		(0.08)		(0.07)
Hispanic		0.043		−0.019		−0.147^*^		0.052		−0.001		0.465^***^		0.296^**^
		(0.03)		(0.03)		(0.07)		(0.08)		(0.02)		(0.12)		(0.11)
Other		−0.022		−0.038		−0.204^**^		−0.102		−0.019		0.398^**^		0.364^**^
		(0.03)		(0.03)		(0.07)		(0.08)		(0.03)		(0.13)		(0.12)
Experience		−0.001		0.001		0.002		−0.002		0.001		−0.013^**^		−0.004
		(0.00)		(0.00)		(0.00)		(0.00)		(0.00)		(0.00)		(0.00)
Education		0.006		0.008		−0.005		0.017		0.013^***^		−0.103^***^		−0.074^***^
		(0.00)		(0.00)		(0.01)		(0.01)		(0.00)		(0.02)		(0.02)
Number of staff		0.017^*^		0.004		0.042^*^		0.000		0.010		−0.070^*^		−0.069^*^
		(0.01)		(0.01)		(0.02)		(0.02)		(0.01)		(0.03)		(0.03)
One session		0.006		0.015		0.080		0.067		0.011		0.041		−0.034
		(0.02)		(0.02)		(0.04)		(0.05)		(0.02)		(0.07)		(0.07)
Enrollment/session		0.002^*^		0.001		0.005^*^		0.003		0.001		0.006		−0.003
		(0.00)		(0.00)		(0.00)		(0.00)		(0.00)		(0.00)		(0.00)
IEP		−0.099^**^		0.016		−0.230^**^		−0.240^**^		−0.002		0.036		0.239
		(0.03)		(0.03)		(0.08)		(0.09)		(0.03)		(0.13)		(0.12)
Low income		0.050^**^		0.032^*^		0.125^**^		0.189^***^		0.024		−0.132^*^		−0.265^***^
		(0.02)		(0.01)		(0.04)		(0.04)		(0.01)		(0.07)		(0.06)
English only		0.037		0.022		−0.040		−0.080		0.012		−0.319^***^		−0.374^***^
		(0.02)		(0.02)		(0.05)		(0.06)		(0.02)		(0.09)		(0.08)
EC certification		-0.035		0.000		−0.000		−0.112^*^		−0.022		−0.101		−0.126
		(0.02)		(0.02)		(0.05)		(0.05)		(0.02)		(0.08)		(0.07)
Other certification		−0.045^*^		−0.019		−0.042		−0.040		−0.009		0.028		−0.114
		(0.02)		(0.02)		(0.05)		(0.06)		(0.02)		(0.09)		(0.08)
Midwest		0.008		0.001		−0.004		0.111		0.018		−0.134		0.073
		(0.02)		(0.02)		(0.05)		(0.06)		(0.02)		(0.09)		(0.08)
South		0.017		−0.017		0.092		0.082		−0.008		0.157		0.035
		(0.02)		(0.02)		(0.05)		(0.06)		(0.02)		(0.08)		(0.08)
West		−0.044		−0.011		−0.013		0.009		0.008		−0.015		0.187^*^
		(0.02)		(0.02)		(0.05)		(0.06)		(0.02)		(0.09)		(0.09)
Constant	4.892^***^	4.790^***^	4.911^***^	4.825^***^	4.365^***^	4.216^***^	4.370^***^	4.182^***^	4.899^***^	4.778^***^	3.848^***^	4.531^***^	2.828^***^	3.554^***^
	(0.01)	(0.04)	(0.01)	(0.04)	(0.03)	(0.10)	(0.03)	(0.12)	(0.01)	(0.04)	(0.05)	(0.17)	(0.05)	(0.16)
*N*	2644	2644	2645	2645	2643	2643	2622	2622	2644	2644	2641	2641	2644	2644
*R-squared *_*adj*_	0.005	0.021	0.006	0.010	0.030	0.046	0.011	0.026	0.008	0.016	0.015	0.117	0.099	0.167

*p < 0.05, **p < 0.01, ***p < 0.001

Program auspice continued to be a significant predictor of curriculum integration and the amount of whole group time in logistic regressions controlling for other factors (Tables 4 and 5). Both public school and Head Start teachers exhibited higher rates of integration of subject matter compared to private teachers in the full models. For whole group time, once teacher and other program characteristics were added into the model, public school was no longer a significant predictor of this practice whereas Head Start remained statistically significant (odds ratio = 0.53, *p* < 0.001). Results were consistent across both measures of auspice.

### Association of practices with teacher and program characteristics other than auspice

Several teacher and program variables were significantly associated with practice measures broadly when also controlling for auspice. Results for these variables were virtually identical comparing analyses using the two alternative measures of auspice. Teacher characteristics most often predicted the use of flashcards and worksheets but sometimes predicted other practices. White non-Hispanic teachers used flashcards and worksheets less often than Black, Hispanic, and Other teachers. Teachers education level was positively associated with choice time and integration of curriculum and negatively associated with the use of flashcards and worksheets. Teachers who spoke only English were less likely to use flashcards and worksheets. Classroom characteristics also predicted practice. The number staff in the classroom besides the teacher was negatively related to use of flashcards and worksheets and positively related to block play and science exploration. Having students who were on average low-income was associated more play-based, child-centered practice on six measures. The higher the percentage of students with an IEP the less frequent was choice for block play and science exploration and exposure to the child’s culture and language. Region generally did not predict practice. However, teachers in the West reported less frequent block play and more use of worksheets, and teachers in the South reported daily time in whole group activities.

## Limitations

Our study has several limitations that should be considered when interpreting the results. First, the data are from 2010. We do not know of any more recent national data on preschool teacher practices. However, our data remain relevant as the academization of preschool practice is a longstanding issue that predates our data, and concerns have not abated more recently (Justice et al., 2020; Stipek, 2006). A study of Head Start found that practices became more academic between 2001 and 2007 but changed little from 2007 to 2015 (Markowitz & Ansari, 2020). One recent study and its literature review suggests substantial continuity in the time allocated to whole group activities and the extent to which activities are child-centered from the time of our data collection to the present. Moreover, the timing of our data is particularly relevant to current debates because it is contemporaneous with the most recent Early Childhood Longitudinal Study kindergarten data that evidenced a decline in developmentally appropriate practice in kindergarten from earlier years and with the Tennessee preschool study cohorts that are alleged to have been harmed by inappropriate practices in the public schools. Finally, there have been no major policy changes in the United States that could be expected to reverse the patterns of practices our study found across auspices (Friedman-Krauss et al., 2022a, 2022b; Friedman-Krauss, Barnett, & Duer, 2022).

Second, our measures of practice are based on teacher self-report. Remarkably little research has been conducted on the validity of preschool teacher report on practice, though what exists supports their validity (Burts et al., 1990; Debnam et al., 2015). Our results could be subject to some social-desirability bias. However, we find no reason to expect social desirability bias to affect one auspice more than the others. Research on teachers of older children has found teacher self-report to provide valid measures of the types of teaching practices used and content of instruction when not in a high-stakes context (Mayer, 1999; Porter, 2002). These measures also provide only broad indicators of the extent of various practices, and additional precision from direct measurement of time allocated to such activities as worksheets would improve our understanding of what the differences reported in this study mean to children.

Third, the representativeness of our sample is difficult to assess as precise data on the characteristics of lead teachers of 4-year-olds by auspice in 2010 are not available. Comparisons with data on Head Start for multiple years, the 2012 National Survey of Early Care and Education, and other sources suggest that our sample may have somewhat overrepresented teachers with a BA across all auspices (Aikens et al., 2011; Carolan, 2013; National Survey of Early Care & Education Project Team, 2015; Paschall et al., 2020; Whitebook et al., 2014). Our sample may be more representative today than it was at the time, as teacher qualifications have increased in Head Start and state-funded preschool programs (Barnett & Friedman-Krauss, 2016; Garver, 2020).

The potential impacts from over-representation of teachers with higher education levels on our findings and conclusions are quite limited. For some practices, the percentages of teachers reporting the highest frequency across all auspices were so overwhelming that changes in the distribution of teachers would have minor impact. For others—such as use of flash cards and worksheets—our study may slightly understate their use, particularly in Head Start in 2010 (though not now as more Head Start teachers have BA degrees). Most importantly, differences by auspice and other associations in our multivariate analyses do not depend on perfect representation of the population, and the differences in practice by auspice are robust to controls for teacher characteristics and other program features that may have been over- or under-represented in our survey.

Finally, our analyses are descriptive. Our regression analyses that include teacher and program characteristics offer some insights into the potential direct and indirect effects of auspice but these estimates and those for teacher and classrooms characteristics should be interpreted with caution. Our primary goal was to assess the extent to which associations with auspice support claims that public schools are more narrowly academic and to identify associations that program characteristics that might be influenced by public policies. Our analyses may suggest paths through which policies operate (including auspice) but they are not structural models. Nevertheless, the fact that public programs were on every measure less likely to engage in practices identified with academization and developmentally inappropriate practice contradicts the assertion that a movement toward greater public provision would increase such practices.

## Discussion

Preschool teachers reported a mix of practices related to play-based, child-centered, academic drill, and whole group approaches to learning and teaching. Although all practices varied somewhat by auspice, some practices were remarkably uniform across and within all auspices. More than 90 percent of teachers in each auspice reported children very often had opportunities to play with blocks, choose among learning areas and activities, and choose play activities. Large majorities from each auspice also reported that children very often had opportunities to explore science materials and experience their own language and culture, though there was substantial variation in the frequency of these practices. Most teachers did not report using math worksheets regularly, but most did report using flashcards regularly (at least a couple of days per week) or more frequently. These practices were the most variable among teachers. Whole group time also varied, though most teachers reported children spent 30 to 60 min per day in whole group activities.

We found no evidence that public school preschools were more academic than private preschools. To the contrary, teachers in private programs reported more academic, less child-centered approaches compared to public school programs on every measure including whole group time and integrating learning across subject areas in the curriculum. These conclusions do not depend on a specific operational definition of auspice. Particularly strong differences were found for two practices often cited as indicative of inappropriate practice in public school preschools—the use of flashcards and worksheets. Some, but not all, of the difference in practice by auspice can be explained by differences in program features such as public programs having teachers with higher levels of education.

Head Start practice was significantly less academic than private provider practice on all measures and sometimes was significantly less academic than in the public schools. As a federally administered program with a single set of standards, it is perhaps not surprising that child-centered, play-based practice was more uniformly reported by Head Start teachers and that the difference between Head Start and private provider reported practice more often persisted after controlling for teacher and program characteristics. Nevertheless, even within Head Start a higher level of teacher education level is associated with less “inappropriate” practice as other studies have found (Walter & Lippard, 2017).

Practice might be expected to be more variable within the public school sector than in Head Start. Policies differ substantially across states, and school districts can have considerable local autonomy regarding curriculum, professional development, and other program features that might affect practice (Friedman-Krauss et al., 2022a, 2022b). The finding that the association between auspice and practice disappeared more often for public schools than for Head Start when controlling for teacher and program characteristics suggests that the public school “effect” more strongly derives from specific policies such as the requirement that teachers have BA degrees.

## Conclusion

Preschool teacher reports of practice obtained from a national survey contradict claims that children in the United States typically experience high levels of inappropriate, overly academic practices in state and local public preschool programs. This survey broadly represented teachers of 4-year-olds in private, public school, and Head Start programs. In contrast to the other auspices, state-funded preschool programs primarily serve 4-year-olds, though there are exceptions (Friedman-Krauss et al., 2022a, 2022b). Across a broad range of measures, public school preschool—and Head Start— teachers reported more play-based, child-centered activities, more integration of subject matter across areas, less time in whole group activities, and less frequent use of flashcards and math worksheets than did teachers in private preschool programs. Although there was enough variation in teacher reported practice that some public programs might have focused on rote learning with few opportunities for play and exploration, the data are not consistent with widespread use of such an approach in public programs. Of course, even teachers in private providers tended to report more appropriate than inappropriate practice, though a majority of these teachers reported at least sometimes using worksheets which was not true of teachers in public programs.

Our study suggests that public policy influences the types of experiences children have in preschool programs, but not in the ways that some have argued. Public programs are more play-based and child-centered, and they spend less time drilling skills and in whole group activities, compared to private programs. The most uniform and standardized—federal Head Start—was generally the least academic. If current public programs were to shift funding  from public to private programs—through vouchers, for example—it seems likely that children would experience less child-centered, play-based practice. The effects might be quite small with some exceptions such as the use of flashcards and worksheets. Of course, the impacts on children could be more extensive if those two practices are indicators of a broader emphasis on direct instruction in specific skills in ways that we did not measure.

Placing our findings in the context of other studies—including some that are much more recent—suggests the field shift its focus from worries about academization to other issues of the preschool curriculum (National Academies of Sciences, Engineering, and Medicine, 2024). Although some classrooms may be overly academic, this is not the modal or predominant problem seen in classrooms (Denker & Atteberry, 2024). Often a substantial part of the day has no instructional focus, and relatively little time is spent on math and science (Denker & Atteberry, 2024; Nores et al., 2022).

Our findings raise several issues to be addressed by public policy and for further research regarding practice generally, how practices vary with the children served, and what policies might be changed to improve practice. As others have noted, there is a great deal of work to be done to identify stronger connections between the experiences preschool programs provide to young children and persistent positive outcomes (Burchinal et al., 2022). Also, the field may need to be more concerned with incoherence and a lack of alignment from preschool through the early grades that hinders building sustained gains in learning and development than that preschool is being overwhelmed by K-12 practice and policy (Stein & Coburn, 2023).

Two findings regarding differences in practice associated with child characteristics raise questions for research and policy. Programs serving more children with special needs (with an IEP) were more academic and less culturally responsive. Is this necessary or does it reflect a failure to consider the needs of the whole child because of a focus on disability? Programs serving lower-income children were less academic. To what extent do these differences in practice reflect differences in the needs of the children served? Might these differences in practices somehow reflect unduly low expectations for the children that relate to content as well as methods? Research with more focus on the intent and content of teacher–child interactions could contribute to better understanding of practices and how they might be improved.

Several program features regulated by public policy—teacher qualifications, class size, and the number of adults in the classroom were associated with differences in practice. These findings resonate with other findings that structural features are a means to improve educational experiences (e.g., Manning et al., 2019). This suggests that specific public policies can be important influences practice. It also contradicts the notion that such regulations raise costs but do not benefit children. While our measures of both program features and practice are too blunt to provide specific guidance for regulations, the findings suggest that future research with finer-grained measures of teacher characteristics, staffing structures, and practices could be highly informative for public policy.

We recommend the development of new, better sources of information about practice to inform policy. Such studies would improve our knowledge about both children’s experiences and how well specific experiences support their learning, development, and wellbeing. For example, a new nationally representative study of preschool practice could provide much more detail than we have and go well beyond commonly used observation measures such as the Environmental Rating Scales (Vermeer et al., 2016) and CLASS (Pianta et al., 2008). Such a study should collect information on the nature and contexts of children’s activities throughout the preschool day including types of activities and interactions, content, and the richness of content (e.g., Bustamante et al., 2018; Dwyer & Harbaugh, 2020; Pianta et al., 2018; Powell et al., 2008). In addition, measures should be sensitive to differences in activities and interactions among children in the same classroom. New insights might be gained by obtaining children’s perspectives on their experiences as well as teacher perspectives and independent observations. Countries other than the United States might benefit even more from such studies as many have national policies that could be informed.

We have used the term “academization” to roughly characterize practices that in the United States many might also call “developmentally inappropriate.” We recognize that there is controversy regarding the conceptualization and application of DAP (e.g., Lubeck, 1998; Sanders & Farago, 2018) but this term is in common usage and critics of preschool education in the public schools seem to have a sufficiently shared view of DAP to usefully convey both their concerns and the substance of our findings. For those who believe that the principles of DAP generally improve the education of young children, then the differences in practices by auspice we find suggest that public providers better support young children’s learning, development, and wellbeing in the United States, keeping in mind the limitations of our measures.

We reiterate that our findings are no reason to be complacent about practice in any auspice. Our measures are less extensive and precise than we would wish, and what constitutes best practice likely depends on the children and families served, teachers, and broader contexts. A recent National Academy of Sciences, Engineering, and Medicine (2024) report has called for a new vision for curriculum that is more responsive to the needs of today’s children and families and more strongly based on evidence of effectiveness. To facilitate this, the field would benefit from both more research of greater complexity regarding practice with a focus on both the “what” and “how” children experience and how changing these might improve learning and development over the long-term. This will require greater investment in both formal research and just as importantly in early childhood program capacity to pursue their own continuous improvement locally including greater supports for teachers to implement well what they understand to be good for young children (Barnett et al., 2021).

## Data Availability

Data related to this study can be obtained upon request from the authors.
